# Crystal structure, DFT calculations and evaluation of 2-(2-(3,4-dimethoxyphenyl)ethyl)isoindoline-1,3-dione as AChE inhibitor

**DOI:** 10.1186/s13065-018-0442-1

**Published:** 2018-06-25

**Authors:** Erik Andrade-Jorge, José Bribiesca-Carlos, Francisco J. Martínez-Martínez, Marvin A. Soriano-Ursúa, Itzia I. Padilla-Martínez, José G. Trujillo-Ferrara

**Affiliations:** 1 Laboratorio de Investigación en Bioquímica, Sección de Estudios de Posgrado e Investigación, Escuela Superior de Medicina del Instituto Politécnico Nacional, Plan de San Luis y Díaz Mirón s/n Casco de Santo Tomás, 11340 Mexico City, Mexico; 2 Facultad de Ciencias Químicas, Universidad de Colima, Km. 9 Carretera Colima-Coquimatlán, C.P. 28400 Coquimatlán, Colima Mexico; 3 Laboratorio de Investigación en Fisiología, Sección de Estudios de Posgrado e Investigación, Escuela Superior de Medicina del Instituto Politécnico Nacional, Plan de San Luis y Díaz Mirón s/n Casco de Santo Tomás, 11340 Mexico City, Mexico; 4 Laboratorio de Química Supramolecular y Nanociencias, Unidad Profesional Interdisciplinaria de Biotecnología del Instituto Politécnico Nacional, Av. Acueducto s/n Barrio la Laguna Ticomán, 07340 Mexico City, Mexico

**Keywords:** AChE inhibitor, Alzheimer’s disease, Crystal structure, Isoindoline-1, 3-Dione, Kinetic

## Abstract

Dioxoisoindolines have been included as a pharmacophore group in diverse drug-like molecules with a wide range of biological activity. Various reports have shown that phthalimide derivatives are potent inhibitors of AChE, a key enzyme involved in the deterioration of the cholinergic system during the development of Alzheimer’s disease. In the present study, 2-(2-(3,4-dimethoxyphenyl)ethyl)isoindoline-1,3-dione was synthesized, crystallized and evaluated as an AChE inhibitor. The geometric structure of the crystal and the theoretical compound (from molecular modeling) were analyzed and compared, finding a close correlation. The formation of the C6–H6···O19 interaction could be responsible for the non-negligible out of phenyl plane deviation of the C19 methoxy group, the O3 from the carbonyl group lead to C16–H16···O3^i^ intermolecular interactions to furnish *C(9)* and *C(14)* infinite chains within the (− 4 0 9) and (− 3 1 1) families of planes. Finally, the biological experiments reveal that the isoindoline-1,3-dione exerts a good competitive inhibition on AChE (Ki = 0.33–0.93 mM; 95% confidence interval) and has very low acute toxicity (LD_50_ > 1600 mg/kg) compared to the AChE inhibitors currently approved for clinical use.
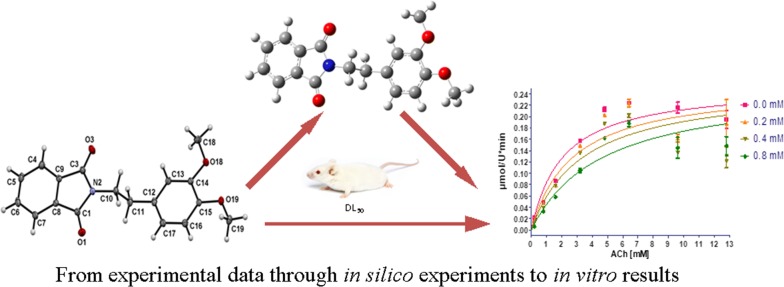

## Introduction

Alzheimer’s disease (AD) is a progressive neurodegenerative disorder. Since the gradual damage to neurons leads to an irreversible deterioration of memory and learning, the afflicted person is eventually unable to carry out cognitive functions [1, 2]. AD is the most common form of dementia in the elderly population [3], accounting for 60–80% of all cases [4–6].

The pathogenesis of AD involves the accumulation of soluble amyloid-β peptide [7], the dysfunction of the cholinergic system, and the deposition of tau neurofibrillary tangles in the brain [8]. These physiological changes lead to confusion, memory loss, impaired cognitive and emotional function, and finally dementia [9].

The main drug target is acetylcholinesterase (AChE) [8], which hydrolyzes the neurotransmitter acetylcholine (ACh) at cholinergic synapses and thus terminates nerve transmission. Since low levels of this signaling molecule are associated with the development of AD, high levels of the same are considered desirable in patients [10–13].

According to the cholinergic hypothesis, impairments in the cholinergic pathway play a pivotal role in the pathogenesis of AD [14]. The main mechanism for enhancing the level of ACh is the inhibition of AChE, which is presently the most effective strategy for treating AD. Hence, the current treatments are cholinesterase inhibitors that target AChE and butyrylcholinesterase (BuChE), and antagonists of *N*-methyl-d-aspartate (NMDA) receptor [1, 2].

In addition to depleting Ach (low concentrations), human AChE accelerates the metabolic rate of formation of the amyloid-β peptide, which exacerbates the clinical progression of AD [15, 16]. Other proteins involved in the development of this disease are tau, α-synuclein and apoE4, and all of them are regulated by the activity of AChE [17]. AChE inhibitors (AChEIs) are the only type of drug approved for the treatment of AD.

The phthalimide ring (isoindoline-1,3-dione) represents an important privileged substructure in diverse molecules exhibiting neuroprotective agents, antioxidant, antihypertensive activity, etc. [18–20]. Numerous reports have identified phthalimide derivatives as potent inhibitors of AChE [21–24] and BuChE [1, 25].

Paneck et al. synthesized and evaluated phthalimide saccharin derivatives, finding one of these to be a selective AChEI that significantly impeded the accumulation of amyloid-β [26]. Simoni et al. developed other new compounds with an indole moiety in their structure that are able to simultaneously inhibit AChE and amyloid-β aggregation [27].

The pharmacophore isoindoline-1,3-dione is known to interact with great affinity at the peripheral anionic site (PAS) of human AChE. To optimize the interaction with the catalytic active site at the same time, the linker between the radical of the drug and the isoindoline-1,3-dione should include an oligomethylene [28].

Hebda et al. described how phthalimide groups interact with the PAS site of AChE. They found that the two carbonyl groups of phthalimide facilitate hydrogen bonding with AChE, and the replacement of phthalimide groups with a heteroaromatic moiety reduces potency [29]. It has also been explained how an electron donating group as a *methoxy* substituent, particularly in the *para* position, confers higher potency to the drug. In the case of electron withdrawing groups, such as chlorine or fluorine moieties, the *ortho* position provides a greater inhibitory effect on AChE [31]. Finally, it was reported how the ability of a ligand to bend (due to alkyl chains) improves its interaction with the anionic and acyl pocket of AChE. Hence, the presence of alkyl chains may be necessary for excellent potency in a competitive or non-competitive inhibitor [30].

Taking into account the above information the compound was design based on the literature, where is described that for good inhibitory effect on AChE the molecule must have an isoindoline group, the presence of 2 carbonyl groups and also the presence of electron donating groups as methoxy moiety, additionally the presences of methylenes are required for good potency. The aim of the current study was to synthesize and crystallize 2-(2-(3,4-dimethoxyphenyl)ethyl)isoindoline-1,3-dione, then compare its molecular X-ray structure with that of the same compound simulated for molecular modeling. Furthermore, its activity as an AChEI was determined in vitro and ED_50_ in vivo.

## Results and discussion

### Molecular Structure

**Fig. 1:**
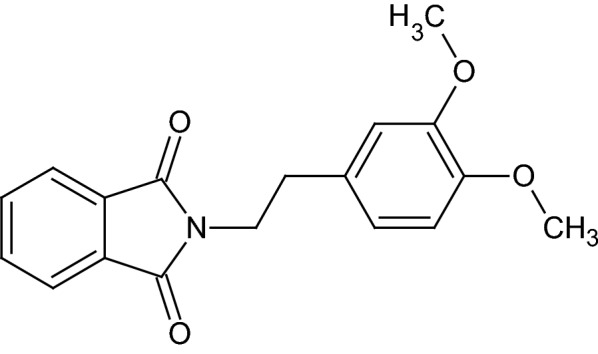
Molecular structure of 2-(2-(3,4-dimethoxyphenyl)ethyl)isoindoline-1,3-dione (**1**)

**Fig. 2:**
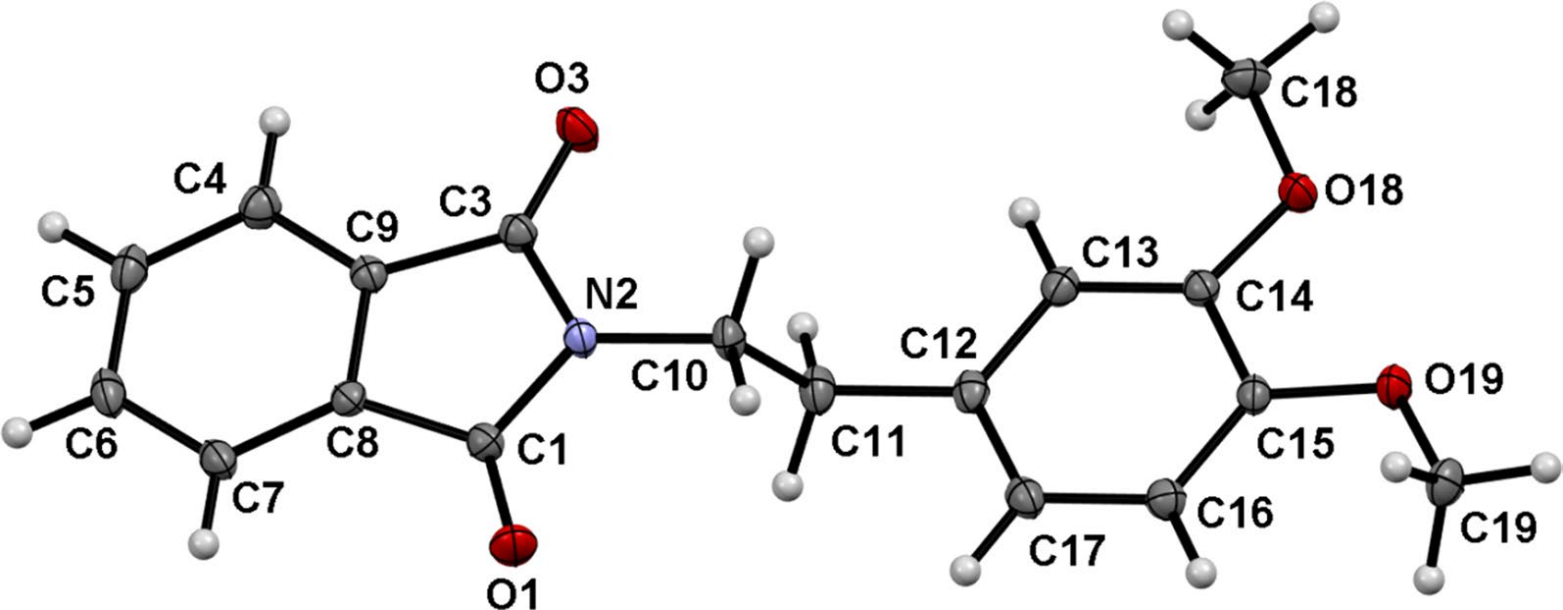
X-ray molecular structure of 2-(2-(3,4-dimethoxyphenyl)ethyl)isoindoline-1,3-dione (**1**) with an atom labeling scheme. ORTEP view at the 50% probability level

The compound 2-(2-(3,4-dimethoxyphenyl)ethyl)isoindoline-1,3-dione (**1**; Fig. 1) was afforded as colorless triclinic crystals in the space group P − 1, with Z = 2. The molecular structure is shown in Fig. 2 and selected bond lengths, bond angles and torsion angles are listed in Table 1. Although the mean value of the N–CO (1.393(6) Å) bond length is longer than the mean value observed in isolated amide group (N–CO=1.325(9) Å), it is within the expected range for imides (1.396(10) Å) [31].

**Table 1:** Comparison between modeled and crystal geometric structures of **1**

	Modeled structure	Crystal structure		Modeled structure	Crystal structure
Energy *(kJ/mol)*	− 2 760 740.50		*E*_*LUMO*_ *(kJ/mol)*	− 549.86	
*E*_*HOMO*_ *(kJ/mol)*	− 777.67		*GAP (kJ/mol)*	− 227.81	
Bond lengths (Å)					
O1–C1	1.239	1.208(2)	N2–C10	1.462	1.456(2)
O3–C3	1.239	1.211(2)	C8–C9	1.404	1.381(2)
N2–C1	1.410	1.397(2)	C14–O18	1.386	1.367(2)
N2–C3	1.409	1.389(2)	O18–C18	1.450	1.427(3)
C1–C8	1.489	1.489(3)	C15–O19	1.387	1.371(2)
C3–C9	1.489	1.484(3)	O19–C19	1.449	1.428(2)
Bond angles (°)					
C9–C3–O3	129.1	129.14(16)	C14–O18–C18	118.4	116.60(15)
O3–C3–N2	124.9	124.64(17)	C16–C15–O19	124.6	124.52(16)
C3–N2–C10	123.8	123.14(14)	C15–O19–C19	118.3	116.51(15)
N2–C10–C11	112.6	111.62(18)	O18–C14–C15	115.8	115.22(16)
C13–C14–O18	124.4	125.17(16)	O19–C15–C14	116.0	116.96(16)
Torsion angles (°)					
O3–C3–N2–C10	1.015	− 3.0(3)	C18–O18–C14–C13	0.271	3.8(3)
O1–C1–N2–C10	− 0.935	1.7(3)	C19–O19–C15–C16	0.258	− 9.2(3)
N2–C10–C11–C12	177.174	179.44(16)	O18–C14–C15–O19	0.038	2.1(3)
C10–C11–C12–C13	− 82.95334	− 102.4(2)			

**Fig. 3:**
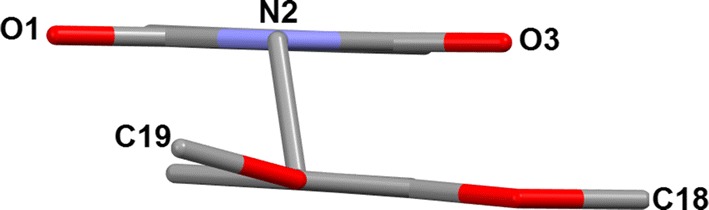
The molecular structure of **1**, viewed along the axis of C11–O19 atoms. The hydrogen atoms are not included for the sake of clarity

The dimethoxyphenyl and isoindoline-1,3-dione rings are almost coplanar with the torsion angles of − 102.4(2)° for C10–C11–C12–C13 and 99.0(2)° for C1–N2–C10–C11. However, the methyl C19 is markedly more twisted than C18. An angle of 3.8(3)° was detected for C18–O18–C14–C13 and − 9.2(3)° for C19–O19–C15–C16 (Fig. 3) these results were confirmed with the theoretical modeling (Table 1).

### Molecular modeling

**Fig. 4:**
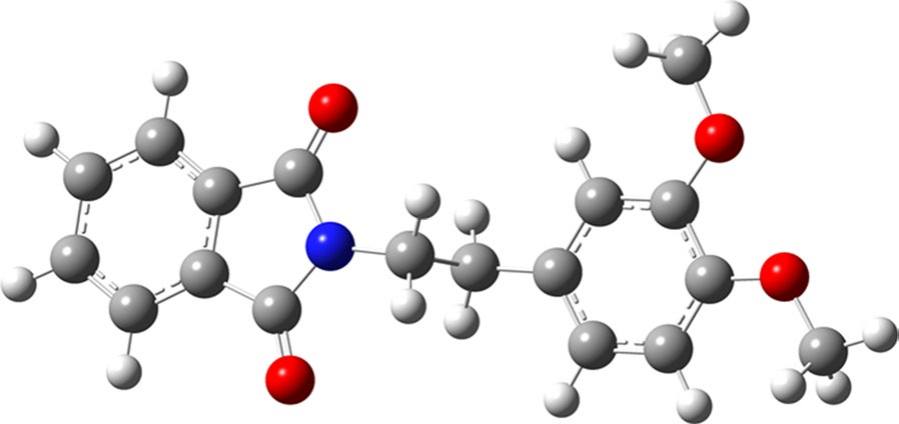
Optimized structure of 2-(2-(3,4-dimethoxyphenyl)ethyl)isoindoline-1,3-dione (B3LYP/6-311G, gas phase)

The DFT calculations showed that the optimized structure for molecular modeling is very similar to the X-ray crystal structure. According to the statistical analysis, there was no significant difference in bond lengths or bond angles between these two structures (two-tailed Student’s *t*-test; p < 0.05). The geometric parameters of the crystal structure and calculations are listed in Table 1. The optimized structure is illustrated in Fig. 4.

### Supramolecular structure

The non-planar arrangement of the C19 methyl may be related to the network arrangement of **1** in the crystal. The geometric parameters associated with intermolecular intermolecular interactions are listed in Table 2. The graph set notation is used to describe the intermolecular interactions motifs [32].

**Table 2:** Geometric parameters of the intermolecular interactions of compound **1**

D–H···A	Symmetry code	D–H (Å)	H···A (Å)	D···A (Å)	D–H···A (°)
C16–H16···O3^i^	x, y − 1, z	0.95	2.58	3.241(3)	127
C6–H6···O19^ii^	x, y + 1, z − 1	0.95	2.58	3.366(2)	140

**Fig. 5:**
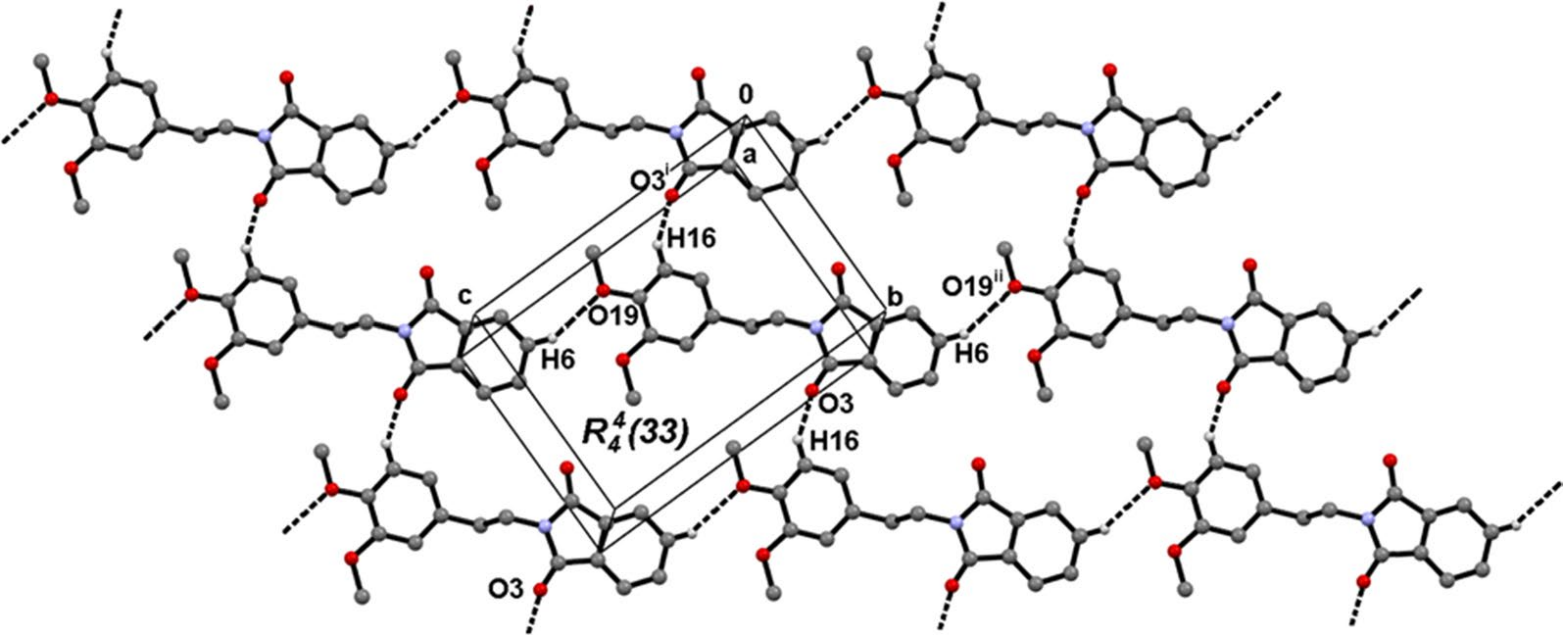
Supramolecular structure of **1**, based on C16–H16···O3 and C6–H6···O19 interactions. Viewed in the *bc* plane. Symmetry codes: (i) x, y − 1, z; (ii) x, y + 1, z − 1

**Fig. 6:**
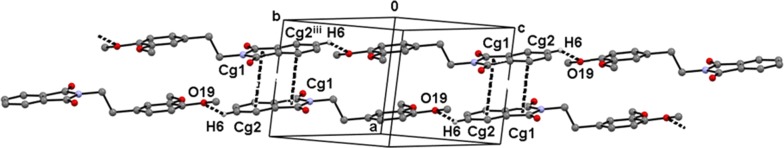
The supramolecular 3D structure of compound **1** based on *Cg1*···*Cg2* interactions along the *a*-axis. Symmetry code: (iii) − 1 − x, 2 − y, − z

The O3 and O19 oxygen atoms, from the carbonyl and methoxy groups, lead to C16–H16···O3^i^ and C6–H6···O19^ii^ intermolecular interactions to furnish *C(9)* and *C(14)* infinite chains within the (− 4 0 9) and (− 3 1 1) families of planes, respectively. Both motifs combined form $R^{4}_{4} \left( {33} \right)$ rings that develop the second dimension in the *bc* plane (Fig. 5). The propagation of π-stacking interactions between the dione (*Cg1* = C1/N2/C3/C8/C9) and fused benzene (*Cg2 *= C4–C9) rings results in *Cg1*···*Cg2*^iii^ stacking (symmetry code iii = 1 − x, 2 − y, − z), which develops the third dimension along the direction of the *a*-axis (Fig. 6). The value of the intercentroid distance between the *Cg1* and *Cg2* rings (3.5364(14) Å) is very close to the value of the interplanar distance (3.4485(10) Å), corresponding to a face-to-face interaction [33], where the dione ring acts as the acceptor of electronic density and the benzene fused ring as the donor. The C6–H6···O19 interaction could be responsible for the non-negligible out of phenyl plane deviation of the methoxy group C19.

The absence of strong hydrogen bonding donors results in the participation of only one imide carbonyl in hydrogen bonding. The selective activation of one imide carbonyl group of the *N*-phenethylimides by Brønsted acids, such as BBr_3_ [34], TfOH [35] or organometallics [36], leads to regioselective intramolecular cyclization to deliver tetrahydroisoquinoline derivatives [37, 38]. This selective regiochemical polarization could be involved in the mode of action of compound **1** as an AChEI, as previously proposed [39].

### In vitro experiments to determine AChE inhibition

**Fig. 7:**
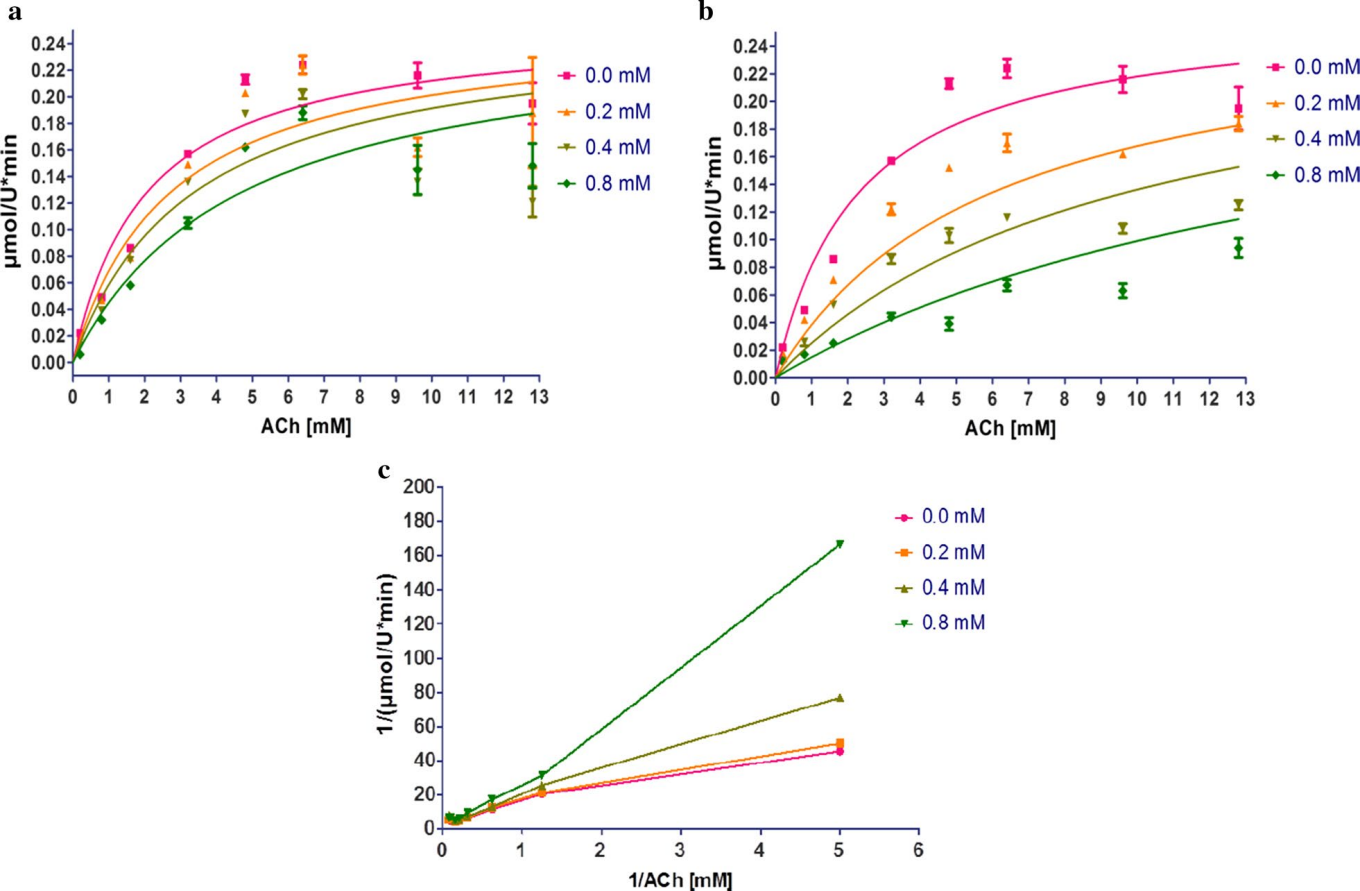
The inhibitory effect on AChE of Electrophorus electricus for: **a** 2-(2-(3,4-dimethoxyphenyl)ethyl)isoindoline-1,3-dione (Ki = 0.33–0.93 mM), and **b** neostigmine as the positive control (Ki = 0.093–0.157 mM; non-linear regression with 95% confidence intervals). **c** Lineweaver–Burk plot for 2-(2-(3,4-dimethoxyphenyl)ethyl)isoindoline-1,3-dione

An in vitro assay was performed to examine the inhibitory effect of the crystallized compound on AChE. The test compound behaves as a competitive inhibitor (Fig. 7), with an inhibitory activity slightly weaker than that of neostigmine. Acute toxicity, examined in CD1 male mice by Lorke’s method (Table 3), proved to be very low (LD_50_ > 1600 mg/kg) compared to other AChEIs approximately from 43- to 3000-fold less toxic that is the case of Neostigmine LD_50_ = 0.54 ± 0.03 mg/kg. The results clearly show that the synthesized compound has very low toxicity compared to the drugs currently on the market, which allows us to propose this molecule as a leader to generate a more potent family of drugs with a low toxicity unlike the drugs currently used for the treatment of AD that has many side effects. Due to the multiple undesirable effects of drugs currently employed to treat AD [1, 2], the present values of 2-(2-(3,4-dimethoxyphenyl)ethyl)isoindoline-1,3-dione suggest the importance of future studies on this and other structurally related compounds to analyze their selectivity for and interactions with cholinesterases, and their potential therapeutic use in the treatment of AD [10, 40].

**Table 3:** Well-known AchE inhibitors with the respective LD_50_ in comparison with compound **1**

Inhibitors	Compound	LD_50_ (mice)
	1	> 1600 mg/kg
	Donepezil	30 mg/kg [41]
	Physostigmine	3 mg/kg [42]
	Neostigmine	0.54 ± 0.03 mg/kg [1]
	Pyridostigmine	37.5 mg/kg [41]

## Conclusion

In summary, the crystal of 2-(2-(3,4-dimethoxyphenyl)ethyl)isoindoline-1,3-dione was obtained and analyzed by x-ray crystallography to determine its geometric structure, which was compared to the optimized structure predicted in the in silico experiment. No significant difference existed between these two structures (experimental and computational modeling) when comparing bond lengths or bond angles. Furthermore, an interesting crystalline network was formed by hydrogen bonding acceptors and soft-hydrogen bonding donors, as well as by dispersive π–π interactions. Finally, an evaluation was made of the inhibitory effect of 2-(2-(3,4-dimethoxyphenyl)ethyl)isoindoline-1,3-dione on AChE, finding a competitive inhibition with a Ki of 330–930 µM (95% confidence interval). The acute toxicity is far less (LD_50_ > 1600 mg/kg) than that of AChE inhibitors currently on the market almost 3000-fold less toxic than Neostigmine LD_50_ = 0.54 ± 0.03 mg/kg. Therefore, future studies are needed to explore the inhibitory activity of this and related isoindoline-1,3-dione derivatives.

## Experimental

### Instrumental

All reagents and solvents were used as received from the commercial supplier (Sigma-Aldrich). All reactions were carried out in an oven-dried flask, agitating the mixtures with a stirring bar and concentrating them with a standard rotary evaporator. The melting point was measured in open-ended capillary tubes with a Stuart^®^ SMP40 automatic melting point apparatus, and is uncorrected. Infrared (IR) spectra were obtained on a 100 FT-IR spectrometer (Perkin-Elmer) with a universal ATR accessory. Thin layer chromatography was performed on 0.25 mm thick silica gel 60 F254 plates (Merck, Darmstadt, Germany) and spots were detected under UV light. ^1^H and ^13^C nuclear magnetic resonance (NMR) spectra were recorded on a Varian Mercury 300 spectrometer (^1^H, 300 MHz; ^13^C, 75 MHz) with tetramethylsilane (TMS) as internal reference. Chemical shifts (δ) are expressed in parts per million (ppm). Other parameters contemplated were the integration area, multiplicity (s = singlet, d = doublet, t = triplet, q = quartet, m = multiplet), and coupling constant (Hz). Electrospray ionization (ESI) high-resolution mass spectrometry was performed on a Bruker micrOTOf-Q-II instrument.

### Chemical synthesis and crystallization

2-(2-(3,4-dimethoxyphenyl)ethyl)isoindoline-1,3-dione was synthesized by employing a reported procedure with slight modifications [43]. In brief, 491 mg (1.50 mmol) phthalic anhydride and 244 mg (1.00 mmol) 2-(3,4-dimethoxyphenyl)ethylamine were mixed and placed into a 50 mL round-bottom flask, then stirred and heated to gentle melting at 150–200 °C for 15–20 min until a dark-yellow color appeared. The reaction was cooled to room temperature and monitored by TLC (using ethyl acetate:hexane in an 8:2 proportion as eluent) before adding 40 mL ethyl acetate and sonicating the reaction to achieve complete dissolution. After the mixture was placed in a separation funnel, 50 mL of water (pH 13) were added (three times) to eliminate the excess of phthalic anhydride. The ethyl acetate was recovered and enough Na_2_SO_4_ and activated carbon were added to be able to filter the mixture. Finally, the solvent was evaporated under a vacuum and the product was recrystallized four times in CH_2_Cl_2_ solution to obtain 0.301 g of colorless block-like crystals (suitable for X-ray) in 90% yield, m.p. = 171–172 °C; IR (ATR, cm^−1^) ύ: 3063 (C–H, Aromatic), 2943 (C–H, Aliphatic), 2842 (O–CH_3_, Aliphatic), 1705 (C=O), 1600 (C=C), 1466 (CH_2_), 1427 (CH_3_), 1394 (C–N), 1228 (O–CH_3_). ^1^H NMR (CDCl_3_, 300 MHz) δ 2.93 (t, H-11), 3.90 (t, H-10), 3.80 (s, H-19), 3.83 (s, H-18), 6.78 (m, H-13,16,17), 7.70 (m, H-5,6), 7.82 (m, H-4,7); ^13^C NMR (CDCl_3_, 75 MHz) δ 168.2 (C-1,3), 123.19 (C-4,7), 132.0 (C-5,6), 130.4 (C-8,9), 39.3 (C-10), 34.0 (C-11), 133.9 (C-12), 111.1 (C-13), 148.7 (C-14), 147.6 (C-15), 111.8 (C-16), 120.8 (C-17), 55.7 (C-18,19). ESI (m/z): 334.0956 [M+Na] [43].

### X-ray diffraction methods

Single-crystal X-ray diffraction data was recorded on a D8 Quest CMOS (Bruker, Karlsruhe, Germany) area detector diffractometer with Mo K α radiation, λ = 0.71073 Å. The structure was solved by using direct methods in the SHELXS97 [44] program of the WinGX package [45]. The final refinement was performed by the full-matrix least-squares method on F^2^ on the SHELXL97 program. H atoms on C were geometrically positioned and treated as riding atoms, with C–H = 0.93–0.98 Å, and Uiso(H) = 1.5 Ueq(C). The Mercury program was utilized for visualization, molecular graphics and analysis of crystal structures [46]. Material was prepared for publication with PLATON software [47]. The crystallographic data were deposited with the Cambridge Crystallographic Data Centre (CCDC) as supplementary publication CCDC number 1563664. Copies of the data can be obtained free of charge upon request from the CCDC, 12 Union Road, Cambridge CB2 1EZ, UK, (Fax: +44-01223-336033 or E-Mail: deposit@ccdc.cam.ac.uk).

Crystal data for C_18_H_17_NO_4_ (M = 311.3 g/mol): triclinic, space group P − 1 (No. 2), a = 7.4363(4) Å, b = 8.7363(4) Å, c = 12.1212(5) Å, α = 89.573(2), β = 80.073(2), γ = 74.650(2)°, V = 747.40(6) Å^3^, Z = 2, T = 163(2) K, Dcalc = 1.38 g/cm^3^, 16,483 reflections measured (2.4° ≤ 2Θ ≤ 25.5°), and 2750 unique (Rint = 0.088, Rsigma = 0.0561) were used in all calculations. The final value of R1 was 0.049 (I > 2σ(I)) and of wR2 0.135 (for all data), GooF = 1.058 and Abs. coefficient = 0.098, min/max (eÅ^−3^), and ΔF = 0.249/− 0.302.

### Molecular modeling

The optimization and vibrational frequency calculations were performed on Gaussian 09 software [48] with the DFT: B3LYP/6-311G basis set.

### In vitro experiments on AChE inhibition

AChE inhibition was evaluated for compound **1** and a known inhibitor, neostigmine, employing the colorimetric method reported by Bonting and Featherstone [49], with a few modifications. This method determines the remaining amount of ACh by measuring the formation of hydroxamic acid from the choline ester after incubation with the enzyme. The color produced by the reaction with acid ferric chloride is related to enzymatic activity, the value of which was established by fitting the data to a typical curve (Fig. 7).

Briefly, Electrophorus electricus was the source of AChE (Sigma Chemical Co. C1682) for the assay. A mixture was made with 0.1 M buffer (pH 8), 0.2 units of AChE, and increasing concentrations of ACh iodide (0.2, 0.8, 1.6, 3.2, 6.4, 9.6 and 12.8 mM) as the substrate for the enzymatic reaction, and 20 min later the alkaline hydroxylamine reagent was added. The test or reference compound was placed in the assay solution (at 0.2, 0.4 or 0.8 mM) and incubated with the enzyme for 20 min at 37 °C. Subsequently, addition was made of the alkaline hydroxylamine reagent and finally the FeCl_3_ reagent. The changes in absorbance at 540 nm were recorded following 10 min of incubation in a Benchmark BIO-RAD. To exclude interference due to the effects of the reference solution, the parameters were determined with the blank, which was the same volume of solution with the drugs, buffered reagents and the enzyme but without acetylthiocholine. The reaction rates were compared, and the inhibition in the presence of the test compounds was calculated. The Ki of each AChE-inhibitor was estimated by using a curve constructed with the steady-state enzyme inhibition constants.

### In vivo experiment (Lethal doses 50) on mice

Briefly, three different groups of 3 (CD1 male mice 20–25 g) were formed, after that each group received one established concentration that was 10, 100 and 1000 mg/kg of our tested compound to determine a range of toxicity. They were observed by 24 h, without presenting toxicity. After that we formed 3 new groups that were used to opening more of the dose spectrum based on first results, this was to probe new doses 1200, 1400 and 1600 mg/kg [50, 51].
